# Gender differences in changes in alcohol consumption achieved by free provision of non-alcoholic beverages: a secondary analysis of a randomized controlled trial

**DOI:** 10.1186/s12889-024-17645-4

**Published:** 2024-01-10

**Authors:** Shohei Dobashi, Kyoko Kawaida, Go Saito, Yukiko Owaki, Hisashi Yoshimoto

**Affiliations:** 1 Institute of Health and Sports Science, University of Tsukuba, Tsukuba, Japan; 2 Research and Development Center for Lifestyle Innovation, University of Tsukuba, 1-2 Kasuga, 305-8550 Tsukuba, Ibaraki Japan; 3 Department of General Medicine and Primary Care, Institute of Medicine, University of Tsukuba, Tsukuba, Japan

**Keywords:** Non-alcoholic beverages, Reduced alcohol consumption, Alcohol drinking behavior, Sex difference

## Abstract

**Background:**

We recently demonstrated that a 12-week intervention consisting of the provision of free non-alcoholic beverages reduced alcohol consumption in excessive drinkers for 8 weeks after the intervention. However, gender differences in this effect were not explored. Thus, this secondary analysis investigated gender differences in the influence of non-alcoholic beverage provision on alcohol consumption.

**Methods:**

Individuals who frequently drank excessively (at least 40 g/day in men and 20 g/day in women) and who were not diagnosed with alcoholism were recruited. Participants were randomized into the intervention or control group by simple randomization using a random number table. In the intervention group, free non-alcoholic beverages were provided once every 4 weeks for 12 weeks (three times in total). The consumption of alcoholic and non-alcoholic beverages was calculated based on a drinking diary submitted with the previous 4 weeks’ of data. In this study, we compared the longitudinal changes in alcohol consumption between genders in both groups.

**Results:**

The provision of non-alcoholic beverages significantly reduced alcohol consumption in both genders; however, significant differences in alcohol consumption between the control and intervention groups were observed only in men. The average alcohol consumption during the intervention fell below the level associated with a high risk of non-communicable diseases in men (32.7 g/day), but not in women (24.8 g/day). Correlation coefficient analysis showed that replacing alcoholic beverages with the provided non-alcoholic beverages resulted in different drinking patterns according to gender. The percent changes in the consumption of alcoholic and non-alcoholic beverages relative to baseline levels did not differ between genders.

**Conclusions:**

Our results suggest that the provision of non-alcoholic beverages reduced alcohol consumption irrespective of gender. Of note, providing non-alcoholic beverages might be particularly useful for reducing high-risk alcohol consumption in male excessive drinkers.

**Trial registration:**

UMIN UMIN000047949. Registered 4 June 2022.

**Supplementary Information:**

The online version contains supplementary material available at 10.1186/s12889-024-17645-4.

## Introduction

Excessive drinking is an important social issue impacting public health worldwide. For instance, chronic excessive alcohol consumption can lead to the development of several conditions, such as high blood pressure, heart disease, stroke, liver cirrhosis, and digestive symptoms, and other serious problems including immune system impairment, learning and memory dysfunction, and mental illness [1]. A recent systematic review and meta-analysis concluded that higher alcohol consumption was significantly associated with all-cause mortality risk [2]. Over the past several decades, many researchers have used animal and human experimental models to investigate methods of reducing the risk of alcohol use disorders [3], but effective approaches have yet to be established.

In this context, we recently conducted a randomized controlled trial showing that a 12-week intervention involving the provision of free non-alcoholic beverages significantly reduced alcohol consumption in excessive drinkers without alcoholism [4]. The trial also showed that the reduction in alcohol consumption was significantly associated with an increase in non-alcoholic beverage consumption. These results suggest that the reduction of alcohol consumption might have occurred due to the replacement of alcoholic beverages with non-alcoholic beverages. Thus, providing non-alcoholic beverages may be an effective strategy to reduce alcohol consumption in excessive drinkers.

However, many previous studies reviewed by Frazen et al. demonstrated that various interventions were associated with individual differences in the effectiveness of reducing alcohol consumption [5]. One of the most representative differences is gender [6, 7]. Previous studies showed gender differences in several neurobiological responses to alcohol consumption [8, 9]. These studies suggested that the increase in blood alcohol concentration in response to drinking was greater in women compared with men, and therefore women might be at greater risk of harmful effects and addiction. Moreover, some studies showed that there were gender differences in the responses to treatment for excessive alcohol consumption [10, 11]. Thus, the effectiveness of reducing alcohol consumption by providing non-alcoholic beverages might differ between men and women. While clarifying this issue may be of great public health importance, it has not yet been investigated.

Therefore, the present study was performed to confirm our hypothesis that gender influences the effect of non-alcoholic beverage provision on alcohol consumption.

## Methods

### Study design, procedure, and measurements

The current study presents expanded data from our recent investigation that examined how the free provision of non-alcoholic beverages impacted alcohol consumption [4]. That study was conducted after approval by the ethics committee of the University of Tsukuba (Notification Number G299), and was registered as UMIN000047949 (Randomized controlled study on the impact of serving non-alcoholic beverages on alcohol consumption). We adopted a single-center, open-label, randomized, parallel-group design. Individuals who drank on 4 or more days per week, with alcohol consumption of at least 40 g for men or 20 g for women on each of those days, were enrolled. The cutoff of this volume of alcohol consumption was medium risk was according to the definition of the World Health Organization [12]. The exclusion criteria were consumption of non-alcoholic beverages at least twice per month, past history of liver disease, current pregnancy or nursing, alcoholism, lack of consent for the use of LINE® (a messaging application adopted widely throughout Japan that can be used on personal computers or smartphones; LINE Corp., Shinjuku-ku, Tokyo, Japan), and inability to understand the study explanation or answer the pre-intervention online survey, both of which were written only in Japanese. This study excluded individuals with alcoholism because it has been suggested that their use of non-alcoholic beverages may enhance alcohol craving and stimulate the desire to drink, which may increase the risk of drinking relapse [13].

At a group briefing before the intervention, written informed consent was obtained from the participants. At the same time, they were asked to complete a questionnaire regarding factors such as age, sex, race, marital status, highest level of education, employment status, household income, smoking history, and subjective view of health, as well as the Alcohol Quality of Life Scale (AQoLS) [14] and questions related to drinking, specifically the number of binge-drinking episodes within the past month and the items in the Alcohol Use Disorders Identification Test (AUDIT) [15]. In addition, height and body weight were measured and a saliva test was administered to assess the activity of genes related to alcohol metabolism, such as alcohol dehydrogenase 1B (*ADH1B*) and aldehyde dehydrogenase 2 (*ALDH2*) [16].

Following the briefing, simple randomization using a random number table was used to randomly allocate the participants to the non-alcoholic beverage provision (intervention) group or to the control group [17]. In the intervention group, free non-alcoholic beverages were provided once every 4 weeks for 12 weeks (three times in total). In this study, we used non-alcoholic beverages that contained 0.00% alcohol by volume, with a taste akin to alcoholic beverages, and designed or suggested for individuals aged 20 years or older as defined by the Japanese Alcoholic Beverage Advertising Review Committee. The beverages were selected depending on each participant’s preferences. Since the purpose of this study was to investigate whether increased availability of non-alcoholic beverages would change the amount of alcohol consumption, there were no stipulations regarding how to drink (amount or frequency). Participants in both groups were asked to record their consumption of alcoholic and non-alcoholic beverages in a calendar-format drinking diary every day, from 2 weeks before the start of the 12-week intervention to 8 weeks after its completion. All participants were also required to submit the drinking diary to the research staff every 4 weeks using LINE®. After the briefing, the study participants were contacted only by phone or via internet. At the end of the study, a gift card worth 10,000 yen (approximately 73.67 US dollars) was given to all participants as a reward. In addition, each participant in the control group received up to five cases of non-alcoholic beverages of their choice.

### Data analysis

**Fig. 1:**
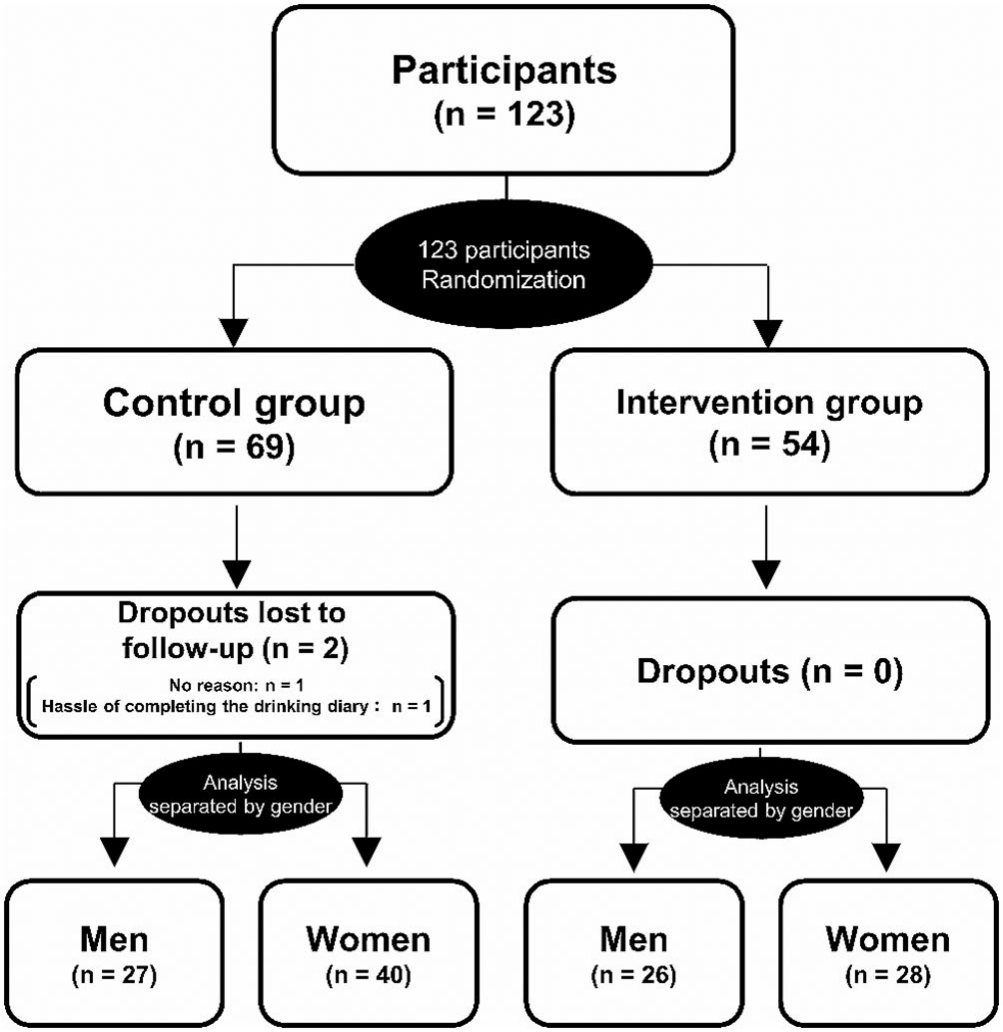
Study flow diagram

Although the amounts of alcoholic and non-alcoholic beverages consumed were obtained every 4 weeks, for practical purposes the mean amounts of alcoholic and non-alcoholic beverages consumed per day were used in the analyses. We calculated the amount of alcohol consumption from the drinking diary data using the following formula: “consumption (mL) x alcohol concentration (%, v/v) x specific gravity (0.8)/100” [18]. We also calculated drinking frequency (drinking days per 4 weeks), the number of days on which participants consumed either alcoholic or non-alcoholic beverages or both, and alcohol consumption on the day of drinking. In this study, we separately analyzed the measurements by gender, and compared the effect of providing non-alcoholic beverages between men and women (Fig. 1). As a previous study suggested that the absolute amount of alcohol consumption is higher in men than women [7], we expressed changes in alcohol consumption during the intervention and follow-up period as percent changes from baseline levels in men and women in the control and intervention groups. Percent changes in non-alcoholic beverage consumption could not be calculated if participants did not consume non-alcoholic beverages at baseline. Hence, the change from baseline in the consumption of non-alcoholic beverages was calculated based on the amount (mL) of non-alcoholic beverages consumed.

Furthermore, to examine whether alcohol consumption was reduced by replacing alcoholic beverages with non-alcoholic ones, the correlation between changes in alcohol consumption and the consumption of non-alcoholic beverages was analyzed.

### Statistical analysis

The normality of the data was evaluated by the Kolmogorov–Smirnov test. Intergroup comparisons of baseline data in each gender were performed by the t-test in cases of normal distribution, and by the Mann–Whitney U test in cases of non-normal distribution. The chi-square test or Fisher’s exact probability test was used to compare categorical variables. Two-way analysis of variance (ANOVA) was used to analyze longitudinal changes in the consumption of alcoholic and non-alcoholic beverages in the two groups separated by gender, using group and time as factors. Using group and gender factors, two-way ANOVA was also used to analyze changes in the consumption of alcoholic and non-alcoholic beverages in men and women during the study period, including the intervention and follow-up period, relative to baseline levels. Normality of the data was not observed for either variable. However, homogeneity of the variance of the data was confirmed for both variables using Levene’s test, and therefore the results of the analysis of variance were accepted with reference to a preceding study [19]. The post hoc Bonferroni’s test was performed to analyze time course changes within a single group, as well as intergroup differences at individual time points or gender differences in the same group. Correlations were evaluated using Spearman’s rank correlation coefficient. The significance level was 5%. The effect size of the two-way ANOVA was described as partial η squared (*η*_p_^2^). GraphPad Prism v. 9.0 (GraphPad Inc., La Jolla, CA, USA) and Stata 18 SE for Windows (Stata Corp., College Station, TX, USA) were used for all analyses.

## Results

One-hundred twenty-three people participated this study and all of them were randomized. After the randomization, we confirmed that there were no significant differences in basic attributes, as previously shown in our original study [4]. The baseline characteristics of participants stratified by gender are shown in Table 1. For both genders, there were no significant differences between the control and intervention groups in age, height, body weight, AUDIT score, number of binge-drinking episodes, number of episodes of heavy episodic drinking, AQoLS score, subjective view of health, proportion of individuals who were Japanese, marital status, highest level of education, employment status, household income, or smoking history (Table 1 and Table S1). The proportion of participants with polymorphisms of the *ADH1B* and *ALDH2* genes was significantly different between the control and intervention groups in men, but not in women (Table 1).

**Table 1:** Baseline characteristics of participants in this secondary analysis

	Men (*n* = 53)		P-value	Women (*n* = 68)		P-value
	Control (*n* = 27, 50.9%)	Intervention (*n* = 26, 49.1%)		Control (*n* = 40, 58.8%)	Intervention (*n* = 28, 41.2%)	
Age (years, SD)	46.4 (10.8)	47.1 (12.7)	0.846^a^	48.0 (9.1)	48.5 (8.6)	0.878^a^
Height (cm, SD)	174.5 (5.6)	172.5 (5.1)	0.166^a^	159.7 (4.9)	159.1 (4.7)	0.709^a^
Body weight (kg, SD)	74.4 (10.2)	74.2 (9.8)	0.945^a^	57.0 (12.2)	55.0 (9.4)	0.584^c^
Genes involved in alcohol metabolism						
ADH1B (number of participants, %)			0.002^b^			0.933^b^
*1/*1	4 (14.8)	1 (3.8)		4 (10.0)	2 (7.1)	
*1/*2	5 (18.5)	6 (23.1)		11 (27.5)	9 (32.1)	
*2/*2	18 (66.7)	19 (73.1)		25 (62.5)	17 (60.7)	
ALDH2 (number of participants, %)			0.024^b^			0.886^d^
*1/*1	19 (70.4)	25 (96.2)		32 (80.0)	22 (78.6)	
*1/*2	8 (29.6)	1 (3.8)		8 (20.0)	6 (21.4)	
*2/*2	0 (0.0)	0 (0.0)		0 (0.0)	0 (0.0)	
AUDIT (points, median, IQR)	12.0 (8.0)	12.5 (4.5)	0.808^c^	8.0 (5)	8.0 (8.0)	0.364^c^
Binge drinking (times/month, median, IQR)	6.0 (14.0)	5.0 (22.8)	0.814^c^	2.0 (10.0)	4.0 (10.0)	0.694^c^
Number of HED episodes (times/4 weeks, median, IQR)	6.0 (19.0)	5.0 (13.8)	0.870^c^	1.0 (3.0)	1.0 (4.0)	0.601^c^
AQoLS (points, median, IQR)	2.0 (4.0)	3.0 (5.5)	0.566^c^	1.0 (4.0)	3.0 (5.3)	0.138^c^
Subjective view of health (number of participants, %)			0.389^b^			0.319^b^
Very healthy	5 (18.5)	6 (23.1)		10 (25.0)	11 (39.3)	
Fairly healthy	22 (81.5)	18 (69.2)		28 (70.0)	17 (60.7)	
Not so healthy	0 (0.0)	2 (7.7)		2 (5.0)	0 (0.0)	
Not healthy	0 (0.0)	0 (0.0)		0 (0.0)	0 (0.0)	

ADH1B, alcohol dehydrogenase 1B; ALDH2, aldehyde dehydrogenase 2; AUDIT, Alcohol Use Disorders Identification Test; HED, heavy episodic drinking; AQoLS, Alcohol Quality of Life Scale, SD, standard deviation; IQR, interquartile range. ^a^ t-test, ^b^ Fisher’s exact probability test, ^c^ Mann–Whitney U test, ^d^ Chi-square test

**Fig. 2:**
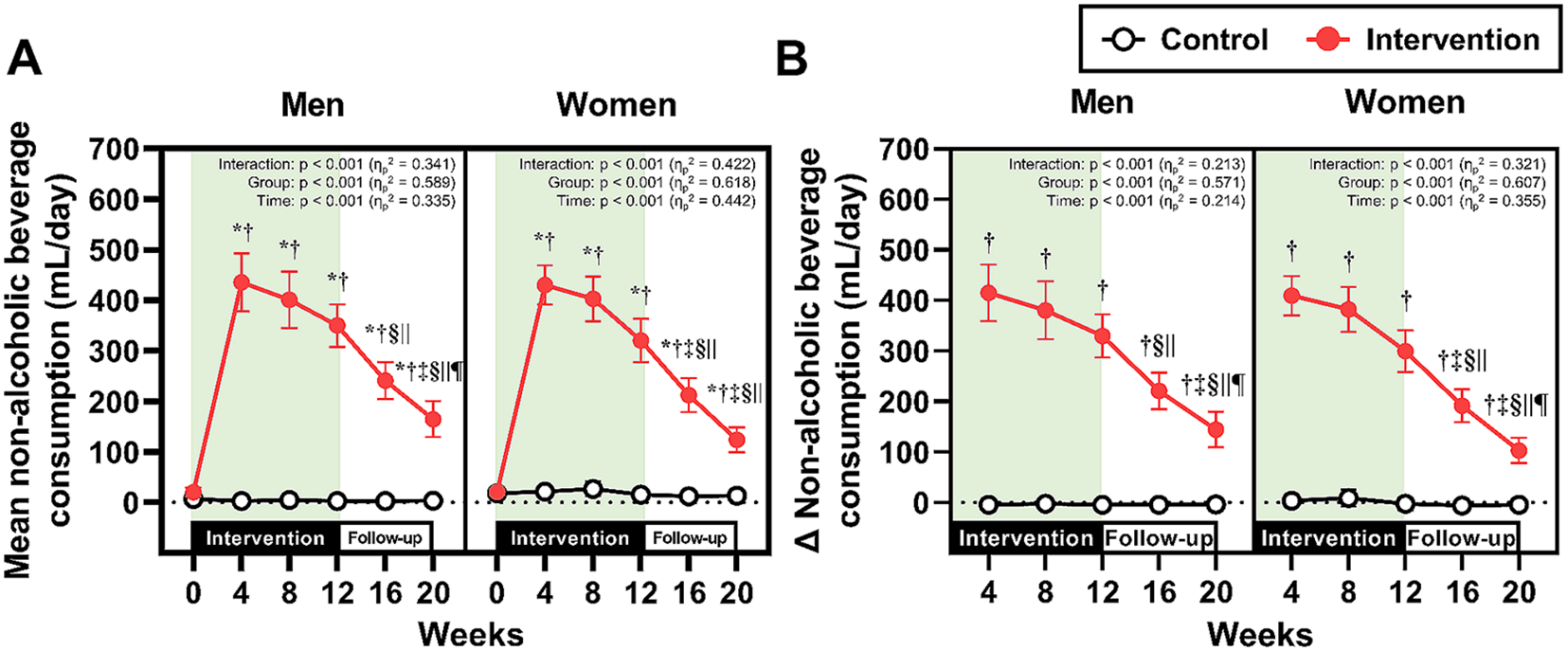
Non-alcoholic beverage consumption. (**A**) Absolute values of mean non-alcoholic beverage consumption throughout the study period in men and women. (**B**) The changes in non-alcoholic beverage consumption from baseline (Week 0) in men and women. *p < 0.05 vs. Week 0, ^†^*p* < 0.05 vs. the control group at the same time point, ^‡^*p* < 0.05 vs. Week 4, §*p* < 0.05 vs. Week 8, ^||^*p* < 0.05 vs. Week 12, and ^¶^*p* < 0.05 vs. Week 16

**Fig. 3:**
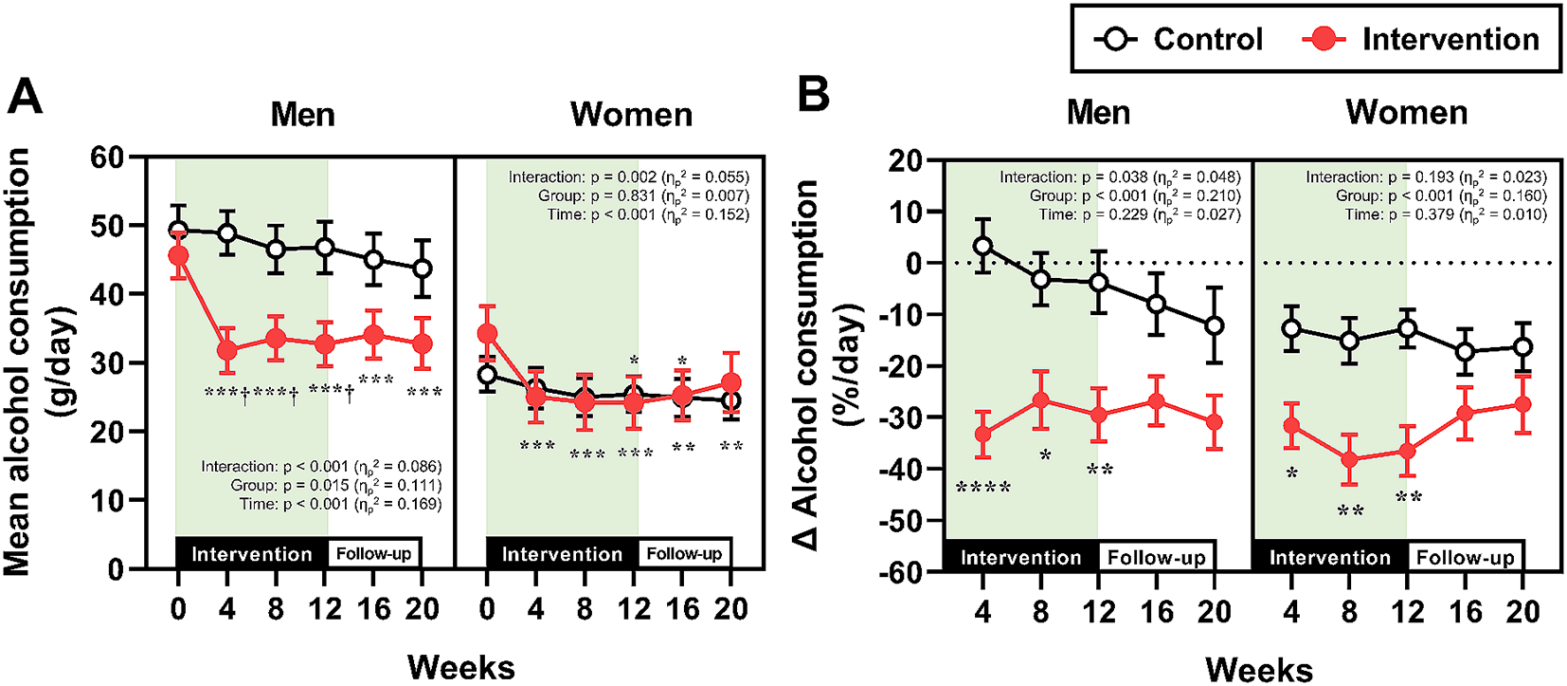
Alcohol consumption. (**A**) Absolute values of mean alcohol consumption throughout the study period in men and women. ***p < 0.001, **p < 0.01, *p < 0.05 vs. Week 0 within the same group. ^†^*p* < 0.05 vs. the control group at the same time point. (**B**) The percent changes in alcohol consumption from baseline (Week 0) in men and women. ****p < 0.0001, **p < 0.01, *p < 0.05 vs. the control group at the same time point

The longitudinal changes in non-alcoholic beverage consumption and alcohol consumption, which were separately analyzed by gender, are presented in Figs. 2 and 3, respectively. In the intervention group, non-alcoholic beverage consumption increased after the start of the intervention in both men and women (Fig. 2A and 2B). Although this consumption gradually decreased after Week 4, it remained significantly greater than that in the control group from Week 4 to Week 20 in both genders. The absolute alcohol consumption in the intervention group was significantly decreased compared with baseline from Week 4 to Week 20, irrespective of gender; however, significant differences between the control and intervention groups were observed only in men during the 12-week intervention (Fig. 3A). The percent changes in alcohol consumption from Week 0 at Weeks 4, 8, and 12 were significantly lower in the intervention group than in the control group in both genders, whereas no significant differences between the two groups were observed during the follow-up period (i.e., Weeks 16 and 20) (Fig. 3B).

**Fig. 4:**
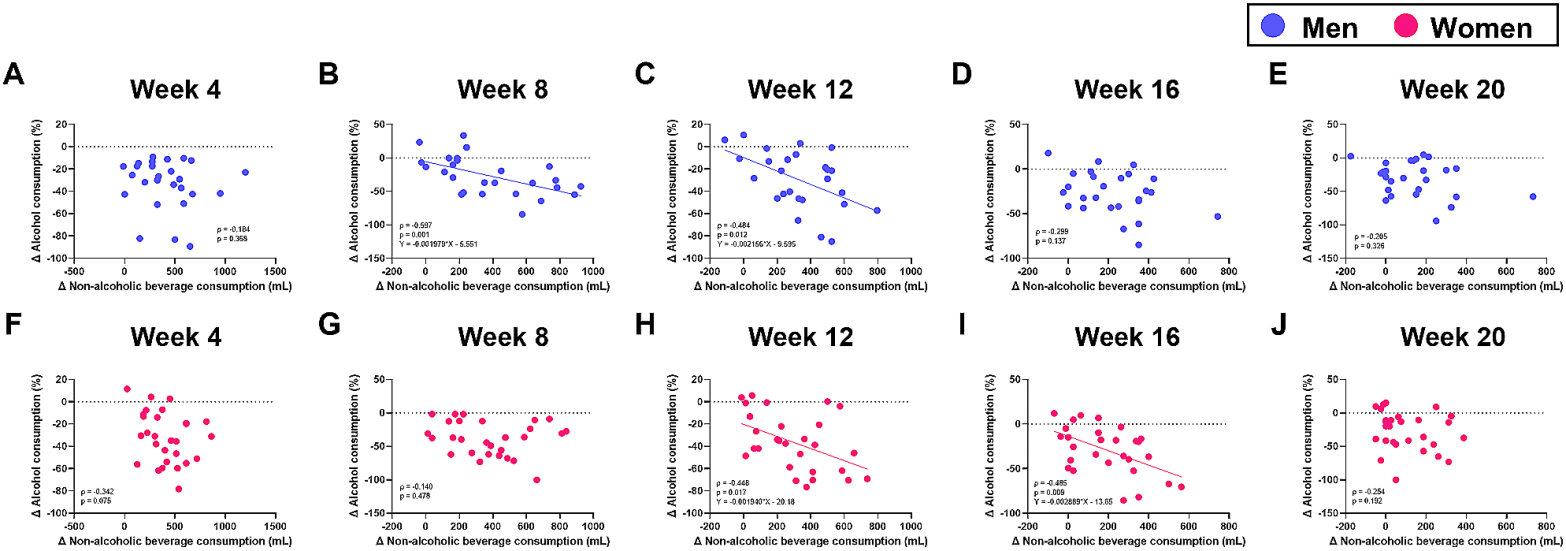
Correlation between changes in alcohol consumption and non-alcoholic beverage consumption at Weeks 4, 8, 12, 16, and 20 in the intervention group. (**A-E**) Men (*n* = 26). (**F-J**) Women (*n* = 28)

Figure 4 shows the correlation coefficients between the percent changes in alcohol consumption and the changes in non-alcoholic beverage consumption at Weeks 4, 8, 12, 16, and 20 in both men and women in the intervention group (Fig. 4). Significant relationships between the changes in alcohol consumption and non-alcoholic beverage consumption were observed at Weeks 8 and 12 in men (Fig. 4B and 4C), but at Weeks 12 and 16 in women (Fig. 4H and 4I).

**Fig. 5:**
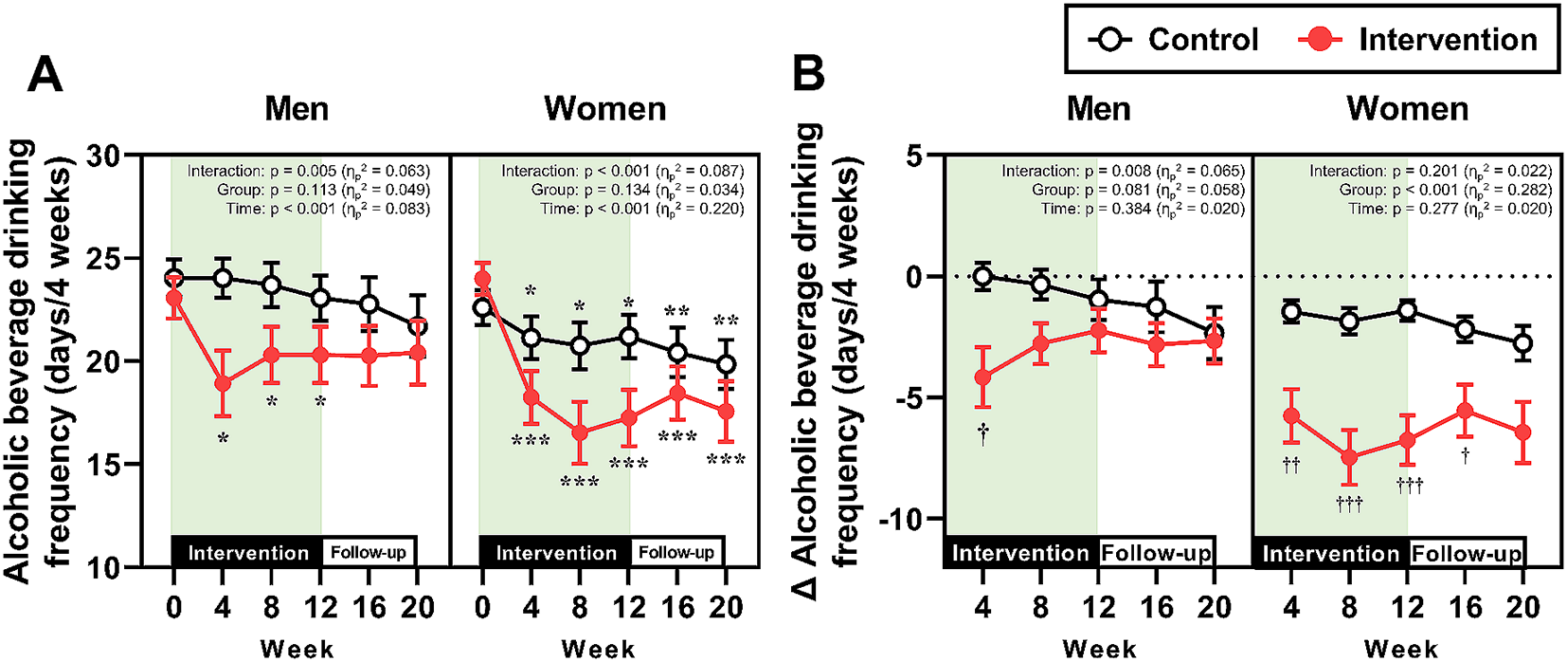
Alcohol beverage drinking frequency. (**A**) Absolute values of mean alcoholic beverage drinking frequency throughout the study period in men and women. ***p < 0.001, **p < 0.01, *p < 0.05 vs. Week 0 within the same group. (**B**) The changes in alcoholic beverage drinking frequency from baseline (Week 0) in men and women. ^†††^*p* < 0.001, ^††^*p* < 0.01, ^†^*p* < 0.05 vs. the control group at the same time point

**Fig. 6:**
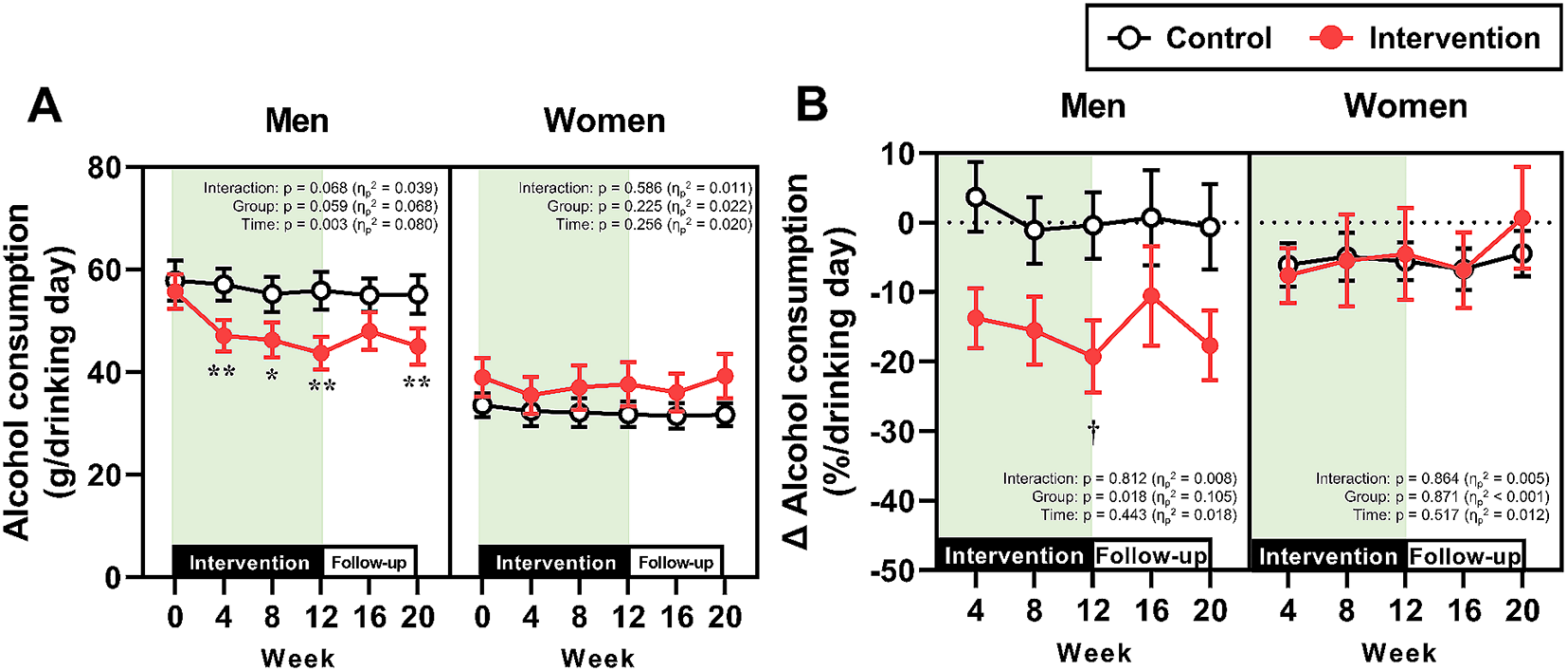
Alcohol consumption on drinking days. (**A**) Absolute values of mean alcohol consumption on drinking days throughout the study period in men and women. **p < 0.01, *p < 0.05 vs. Week 0 within the same group. (**B**) The percent changes in alcoholic beverage drinking frequency from baseline (Week 0) in men and women. ^†^*p* < 0.05 vs. the control group at the same time point

The longitudinal changes in alcoholic beverage drinking frequency and alcohol consumption on the day of drinking, both of which were separately analyzed by gender, are presented in Figs. 5 and 6, respectively. The absolute value of the drinking frequency was significantly lower than at Week 0 in both men and women (Fig. 5A). The reduction in drinking frequency from the values at Week 0 was significant only at Week 4 in men, but from Weeks 4 to 16 in women (Fig. 5B). The absolute values and relative changes of alcohol consumption on drinking days were decreased by the intervention only in men (Fig. 6A and 6B).

**Fig. 7:**
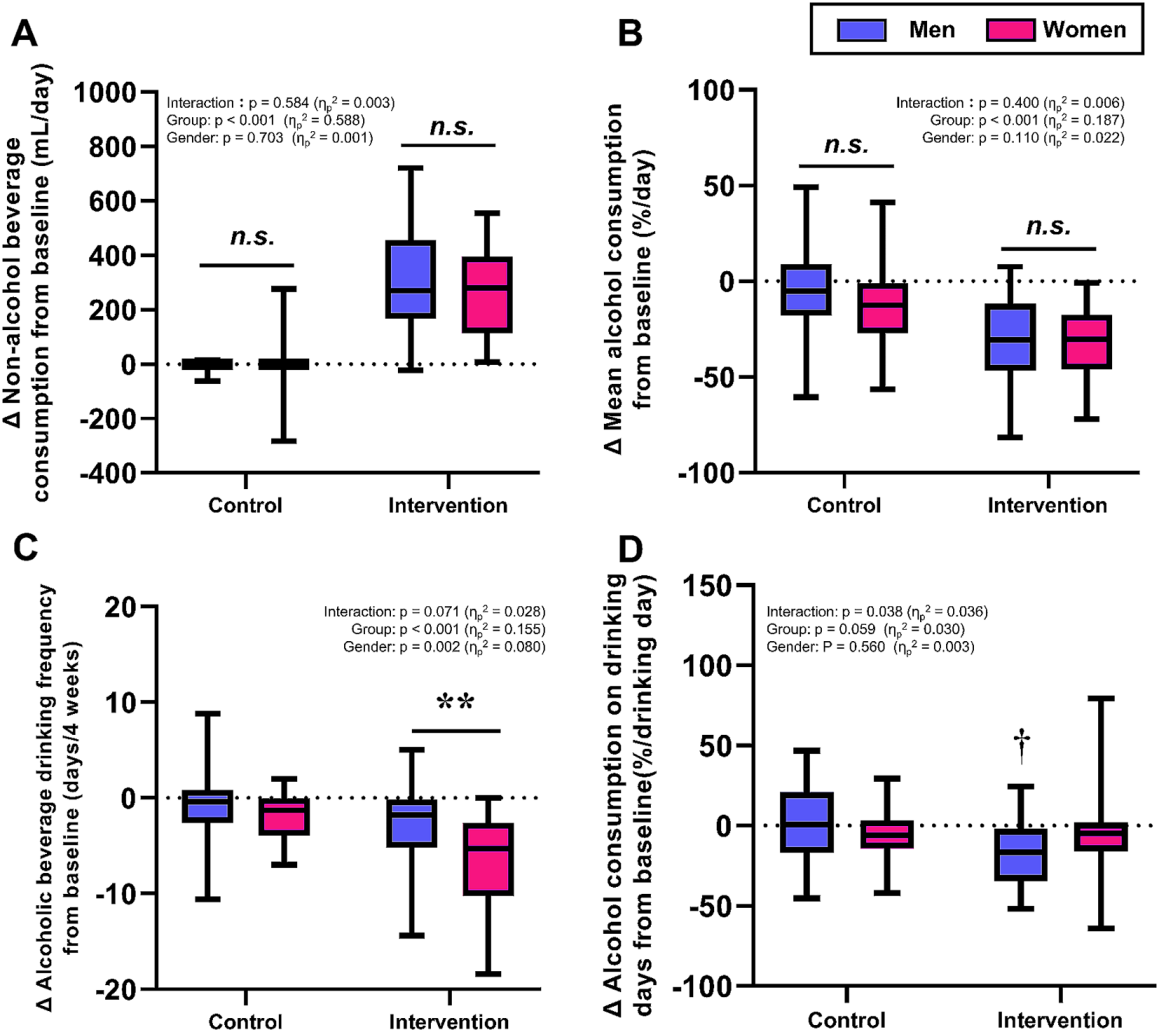
Comparisons between men and women regarding changes from baseline in behaviors related to alcoholic and non-alcoholic beverages. (**A**) Mean non-alcohol beverage consumption, (**B**) Mean alcohol consumption, (**C**) Alcohol beverage drinking frequency, (**D**) Alcohol consumption on drinking day. Each box represents the interquartile range (IQR), with the lower edge of the box indicating the 25th percentile and the upper edge indicating the 75th percentile. The whiskers extend from the box to the minimum and maximum values. *n.s*., not significant. **p < 0.01. ^†^*p* < 0.05 vs. control in men

The mean changes in non-alcoholic beverage consumption, alcohol consumption, drinking frequency, and alcohol consumption on drinking days relative to baseline values throughout the study period are shown in Fig. 7. There was no significant difference between genders in the consumption of either non-alcoholic or alcoholic beverages (Fig. 7A and 7B). In contrast, the change in alcoholic beverage drinking frequency relative to baseline was decreased by the intervention in both genders, and the magnitude was lower in women compared with men (Fig. 7C). Moreover, alcohol consumption on the day of drinking was decreased by the provision of non-alcoholic beverage provisions in men, but not in women (Fig. 7D).

We also counted the number of days on which participants consumed either alcoholic or non-alcoholic beverages or both, and the results are shown in Fig. S1. Irrespective of gender, the provision of non-alcoholic beverages significantly decreased the number of days on which participants drank alcoholic beverages only (Figs. S1A and S1B), and significantly increased the number of days on which they drank non-alcoholic beverages only (Figs. S1D and S1E) or both types of beverages (Figs. S1G-S1H). Relative to baseline values, the number of days on which participants drank both alcoholic and non-alcoholic beverages was marginally higher in men compared with women (Fig. S1I).

## Discussion

In this study, we investigated whether the provision of non-alcoholic beverages differentially affected alcohol consumption in men and women. We found that providing non-alcoholic beverages reduced alcohol consumption in both men and women, but only men exhibited a significant reduction in the absolute amount of alcohol consumption. Moreover, the pattern of replacement of alcoholic beverages with the provided non-alcoholic beverages differed between genders. In contrast, the percent change in alcohol consumption throughout the study period was comparable between genders. These findings suggest that the provision of non-alcoholic beverages may serve as a valuable strategy in public health for reducing alcohol consumption, regardless of gender. However, it is important to note that the effectiveness of this approach differed between genders in some regards.

In this study, a 12-week intervention involving the provision of non-alcoholic beverages significantly increased non-alcoholic beverage consumption until 8 weeks after the completion of the intervention in both genders, which was consistent with our previous results [4]. Although it is well known that the absolute volume of alcohol consumption is higher in men than women [7], the present result suggests that providing non-alcoholic beverages increased their consumption to a similar extent in men and women. However, as we did not distinguish whether the consumed non-alcoholic beverages were provided or voluntarily purchased, we cannot be rule out the possibility that the origin of these beverages differed between genders.

Interestingly, absolute alcohol consumption was significantly reduced in the intervention group compared to the control group in men but not women. We further analyzed the frequency and amount of drinking, and found that men in the intervention group exhibited both a lower drinking frequency and a lower amount on days that they drank, whereas the women in this group demonstrated only reduced drinking frequency. Moreover, the number of days on which participants consumed non-alcoholic beverages was marginally higher in men compared with women. These findings raise the possibility that men who tended to consume non-alcoholic beverages on drinking days were more likely to replace alcoholic beverages with non-alcoholic ones, which may be the reason for the marked reduction in men’s alcohol consumption.

Another possible reason why absolute alcohol consumption was lower in the intervention group compared with the control group in men but not women is that there are gender differences in the likelihood of behavioral changes following the provision of non-alcoholic beverages. A previous study demonstrated that a 3-month online self-help intervention reduced the average number of weekly drinks and the number of drinks per day on which alcohol was consumed in both men and women, but the reduction was significantly greater in men than women [11]. Moreover, Meier et al. [20] simulated the effects of alcohol pricing policies on male and female consumption and estimated that higher prices would lead to a greater reduction of alcohol consumption in men than women. These findings suggest that men are more likely to change their drinking behavior than women. Accordingly, the fact that providing non-alcoholic beverages resulted in a significant reduction in alcohol consumption only in men in this study might be due to gender differences in preferences related to alcoholic beverages and the likelihood of behavioral changes resulting from the intervention to decrease alcohol consumption. However, these explanations are highly speculative, as we did not assess beverage preferences or use any behavioral indices in this study.

The percent changes in alcohol consumption from baseline were significantly lower in the intervention group compared to the control group in both men and women. Moreover, the relative changes in alcohol consumption from baseline throughout the study period did not differ between genders. These results indicate that providing non-alcoholic beverages had a comparable effect on reducing alcohol consumption in men and women. Nevertheless, while our previous study, in which the analysis was not separated by gender, showed a significant reduction in alcohol consumption until 8 weeks after the intervention, [4], the significant reduction disappeared in both men and women in this study. This discrepancy might be due to the small sample size in this secondary analysis. Further studies should be performed in larger populations.

We previously found that the reduced alcohol consumption achieved by providing non-alcoholic beverages might be due to replacing alcoholic beverages with the provided non-alcoholic beverages [4]. Thus, in this study we performed a correlation coefficient analysis of changes from baseline in non-alcoholic beverage consumption and alcohol consumption in the intervention group, with stratification by gender. This analysis showed a significant relationship between non-alcoholic beverage consumption and alcohol consumption at Week 12, and this relationship disappeared at Week 20 in both men and women. Considering that the intervention group had significantly reduced alcohol consumption until Week 20 compared to Week 0, we believe that alcoholic beverages were gradually replaced by non-alcoholic beverages and that a behavioral change may have occurred in these participants to allow them to maintain reduced alcohol consumption without the need for replacement with non-alcoholic beverages. However, a significant relationship between non-alcoholic beverage consumption and alcohol consumption was observed at Weeks 8 and 12 in men, but at Weeks 12 and 16 in women. This result suggests that the altered pattern of alcohol drinking behavior might differ between genders, and the effect of non-alcoholic beverage substitution may occur relatively quickly in men, but a little later in women. In women in the intervention group, the correlation coefficient was highest at Week 16 (i.e., 4 weeks after completing the 12-week intervention). At that time, compared to Week 12, non-alcoholic beverage consumption had significantly decreased, and alcohol consumption had increased. Thus, the disappearance of significant reductions in alcohol consumption during the follow-up period in women might be involved in the lack of non-alcoholic beverage consumption. It is possible that fewer non-alcoholic beverages were consumed during the follow-up period because all of the provided beverages had already been consumed. As such, providing non-alcoholic beverages for longer than 12 weeks may lead to a more effective reduction of alcohol consumption in women. Notably, we also observed that the effects of non-alcoholic beverage provision on drinking frequency and alcohol consumption on drinking days differed between men and women. These findings raise a new hypothesis that the mechanism of reduction in alcohol consumption by providing non-alcoholic beverages differs between genders; specifically, men reduce the amount they drink on a drinking day rather than their drinking frequency, whereas the opposite is true in women. Thus, our current results may provide important evidence for establishing a tailor-made strategy to reduce alcohol consumption in excessive drinkers. Nevertheless, the reason why alcohol drinking behavior varied by gender remains unknown, and future detailed studies are warranted.

Importantly, the mean alcohol consumption during the intervention decreased by approximately 30% in both genders in this study. A previous study indicated that a 30% decrease in alcohol consumption was associated with improved quality of life [21]. Thus, our results suggest that providing non-alcoholic beverages might be a useful public health approach for reducing alcohol consumption, irrespective of gender. Moreover, the average alcohol consumption during the intervention was reduced by 32.7 g/day in men and 24.8 g/day in women. In 2000, the World Health Organization (WHO) reported that the risk of developing non-communicable diseases was increased by alcohol consumption of 40 g or more in men and 20 g or more in women [12]. Considering that this study recruited participants who drank more than these amounts at baseline, a reduction to 40 g or less among men in the intervention group implies that that they were able to move from a high-risk state to a relatively low-risk state, which is considered a clinically significant outcome. Thus, it is suggested that the provision of non-alcoholic beverages reduces alcohol consumption in both men and women but may be a particularly effective option for male excessive drinkers. Nevertheless, the WHO recently stated that “when it comes to alcohol consumption, there is no safe amount that does not affect health” based on previous evidence [22]. Hence, the fact that reduction in alcohol consumption achieved by non-alcoholic beverage provision is important but not yet sufficient to solve alcohol-related health problems. Future studies should investigate the combined effects of non-alcoholic beverage provision and other approaches to reduce alcohol consumption (e.g., face-to-face brief counseling interventions by clinicians, providing information and advice to reduce alcohol use using electrical devices, and creating social and physical environments that reduce drinking occasions and drinks within occasions) on alcohol use behavior and health outcomes [23–25].

There are several limitations to the original randomized controlled study, as previously described [4]. Especially, it should be noted that none of the participants in this study had a history of alcoholism, so it remains unknown whether the results of this study can be applied to individuals with alcoholism or those at high risk of other alcohol-related problems. Moreover, the present study was conducted as a secondary analysis, and therefore the smaller sample size was a major limitation. Unexpectedly, the proportion of individuals with polymorphisms of genes related to alcohol metabolism (i.e., *ADH1B* and *ALDH2*) was significantly different between the control and intervention groups in men. These genotypes affect drinking behavior [16], but no studies have investigated whether the effectiveness of the intervention decreases alcohol consumption in people with different genetic polymorphisms of *ADH1B* and *ALDH2*. Thus, it is currently unknown whether the unexpected significant differences in the proportions of these polymorphisms affect changes in alcohol consumption resulting from the provision of non-alcoholic beverages. However, since future studies may show that this intervention has effects that vary depending on genetic polymorphisms of *ADH1B* and *ALDH2*, caution is necessary when interpreting and applying our results. Finally, based on the fact that there were gender differences in the changes in alcoholic beverage consumption following the provision of non-alcoholic beverages, further investigations are required to examine individual differences in the effectiveness of non-alcoholic beverage provision on reducing alcohol consumption.

## Conclusions

A 12-week intervention consisting of the provision of free non-alcoholic beverages reduced alcohol consumption irrespective of gender. In men, alcohol consumption during the intervention fell below the level defined by the World Health Organization as increasing the risk of non-communicable diseases. These results suggest that providing non-alcoholic beverages may be an effective approach to reducing alcohol consumption in both men and women, particularly in male excessive drinkers.

### Electronic supplementary material

Below is the link to the electronic supplementary material.

Supplementary Material 1: Baseline characteristics not shown in Table 1

Supplementary Material 2: Absolute values, changes from baseline values, and gender differences in alcohol use behavior

## Data Availability

The datasets used and/or analysed during the current study available from the corresponding author on reasonable request.
