# Supplementary material for: Gender differences in changes in alcohol consumption achieved by free provision of non-alcoholic beverages: a secondary analysis of a randomized controlled trial
Source: BMC Public Health. 2024 Jan 10;24:150. doi: 10.1186/s12889-024-17645-4 (PMC10782583; doi:10.1186/s12889-024-17645-4)
Supplement: Supplementary file 1 — Supplementary Material 1: Baseline characteristics not shown in Table 1 [file 12889_2024_17645_MOESM1_ESM.docx]

**Supplemental Table 1. Baseline characteristics not shown in Table 1.**

|  | Men (n = 53) |  | P-value | Women (n = 68) |  | P-value |
| --- | --- | --- | --- | --- | --- | --- |
|  | Control (n = 27, 50.9%) | Intervention (n = 26, 49.1%) |  | Control (n = 40, 58.8%) | Intervention (n = 28, 41.2%) |  |
| Japanese (number of participants, %) | 27 (100) | 26 (100) | N.D. | 40 (100) | 27 (96.4%) | 0.412^b^ |
| Married (number of participants, %) | 21 (77.8) | 19 (73.1) | 0.691^a^ | 30 (75.0) | 18 (64.3) | 0.340^a^ |
| Highest level of education (number of participants, %) | |  | 0.202^b^ |  |  | 0.737^b^ |
| Junior high school | 0 (0.0) | 1 (3.8) |  | 0 (0.0) | 0 (0.0) |  |
| High school | 1 (3.7) | 6 (23.1) |  | 5 (12.5) | 7 (25.0) |  |
| Vocational school | 2 (7.4) | 1 (3.8) |  | 6 (15.0) | 3 (10.7) |  |
| Junior college, specialized  Vocational high school | 1 (3.7) | 1 (3.8) |  | 10 (25.0) | 5 (17.9) |  |
| College | 13 (48.1) | 12 (46.2) |  | 14 (35.0) | 10 (35.7) |  |
| Graduate school | 10 (37.0) | 5 (19.2) |  | 5 (12.5) | 3 (10.7) |  |
| Employed (number of participants, %) | 21 (77.8) | 25.0 (96.2) | 0.491^b^ | 39 (97.5) | 26 (92.9) | 0.564^b^ |
| Household income (number of participants, %) | |  | 0.131^b^ |  |  | 0.213^b^ |
| Less than 1 million yen | 0 (0.0) | 0 (0.0) |  | 0 (0.0) | 1 (3.6) |  |
| 1 to 2 million yen | 0 (0.0) | 0 (0.0) |  | 0 (0.0) | 0 (0.0) |  |
| 2 to 3 million yen | 0 (0.0) | 1 (3.8) |  | 1 (2.5) | 2 (7.1) |  |
| 3 to 4 million yen | 1 (3.7) | 2 (7.6) |  | 4 (10.0) | 2 (7.1) |  |
| 4 to 5 million yen | 6 (22.2) | 2 (7.6) |  | 2 (5.0) | 6 (21.4) |  |
| 5 to 8 million yen | 7 (25.9) | 5 (19.2) |  | 12 (30.0) | 4 (14.3) |  |
| 8 to 10 million yen | 6 (22.2) | 2 (7.6) |  | 10 (25.0) | 7 (25.0) |  |
| More than 10 million yen | 7 (25.9) | 14 (53.8) |  | 11 (27.5) | 6 (21.4) |  |
| Smoking history (number of participants, %) | |  | 0.512^a^ |  |  | 0.734^b^ |
| Smoker | 5 (18.5) | 8 (30.8) |  | 6 (15.0) | 3 (10.7) |  |
| Ex-smoker | 8 (29.6) | 8 (30.8) |  | 14 (35.0) | 8 (28.6) |  |
| Non-smoker | 14 (51.9) | 10 (38.5) |  | 20 (50.0) | 17 (60.7) |  |

N.D., Not detected. ^a^Chi-square test, and ^b^Fisher’s exact probability test.
